# Improved Properties of Poly(3-hydroxybutyrate-*co*-3-hydroxyvalerate) Produced by *Comamonas* sp. EB172 Utilizing Volatile Fatty Acids by Regulating the Nitrogen Source

**DOI:** 10.1155/2013/237806

**Published:** 2013-09-11

**Authors:** Mohd Rafein Zakaria, Hidayah Ariffin, Suraini Abd-Aziz, Mohd Ali Hassan, Yoshihito Shirai

**Affiliations:** ^1^Department of Bioprocess Technology, Faculty of Biotechnology and Biomolecular Sciences, Universiti Putra Malaysia, 43400 Serdang, Selangor, Malaysia; ^2^Department of Process and Food Engineering, Faculty of Engineering, Universiti Putra Malaysia, 43400 Serdang, Selangor, Malaysia; ^3^Department of Biological Functions and Engineering, Graduate School of Life Science and Systems Engineering, Kyushu Institute of Technology, 2-4 Hibikino,Wakamatsu-ku, Kitakyushu, Fukuoka 808-0196, Japan

## Abstract

This study presents the effect of carbon to nitrogen ratio (C/N) (mol/mol) on the cell growth and poly(3-hydroxybutyrate-*co*-3-hydroxyvalerate) accumulation by *Comamonas* sp. EB172 in 2 L fermenters using volatile fatty acids (VFA) as the carbon source. This VFA was supplemented with ammonium sulphate and yeast extract in the feeding solution to achieve C/N (mol/mol) 5, 15, 25, and 34.4, respectively. By extrapolating the C/N and the source of nitrogen, the properties of the polymers can be regulated. The number average molecular weight (*M*_*n*_) of P(3HB-*co*-3HV) copolymer reached the highest at 838 × 10^3^ Da with polydispersity index (PDI) value of 1.8, when the culture broth was supplemented with yeast extract (C/N 34.4). Tensile strength and Young's modulus of the copolymer containing 6–8 mol% 3HV were in the ranges of 13–14.4 MPa and 0.26–0.34 GPa, respectively, comparable to those of polyethylene (PE). Thus, *Comamonas* sp. EB172 has shown promising bacterial isolates producing polyhydroxyalkanoates from renewable carbon materials.

## 1. Introduction

Biobased plastics such as polyhydroxyalkanoates (PHAs) are gaining much interest from the polymer researchers as these materials share the thermal and mechanical properties similar to petrochemical based plastics. A lot of research and development were carried out in order to select suitable microorganisms, to find appropriate substrates from renewable and cheaper carbon source and to improve the fermentation and downstream processes in order to make PHAs more economically viable and competitive, comparable to conventional plastics [1]. One of the limiting factors for the economic production of PHAs is the cost of feedstock, which contributes up to 40% of the total operating costs [2]. Several successful studies have been reported on PHA production from cheap and renewable carbon sources, for example, date syrup, mahua flowers, and palm oil mill effluent (POME) [3–5]. Ntaikou et al. [6] reported that combined biohydrogen and biopolymers were produced from olive oil mill wastewater by two-stage reactor system with poly-3-hydroxybutyrate, P(3HB) yield of 8.94% per dry biomass weight. The use of potential nonrenewable substrates could be useful in reducing the overall PHA production cost. Apart from that, fermentation under nonsterile condition, for example, by using activated sludge treating wastewater, has gained interest recently as this process does not need reactor sterilization, and organic materials in wastewater can be considered a negative cost [7]. P(3HB) homopolymer and P(3HB-*co*-3HV) copolymer are normally the main biopolymer products produced from wastewater. This was because most of organic waste materials were converted into VFA during facultative/anaerobic degradation which is suitable for PHA production [6, 7]. This approach sounds as an environmental and economic promising alternative in reducing total production of PHA.

We have recently reported on poly(3-hydroxybutyrate-*co*-3-hydroxyvalerate) [P(3HB-*co*-3HV)] production from a locally isolated bacterium *Comamonas* sp. EB172, which was successfully isolated from an anaerobic digester treating POME [8–10]. This novel bacterium can accumulate PHA when VFAs from anaerobically treated POME were used as carbon sources [9]. The potential of this microorganism to accumulate PHA in various types of VFA and at different initial medium pH has been tested, and the physical and thermal properties of the polyesters produced from *Comamonas* sp. EB172 had also been reported [10].

Apart from our study, there is limited number of reports on the PHA accumulation by *Comamonas* species. It has been reported that *Comamonas acidovorans* was able to accumulate P(3HB-*co*-3HV) and poly(3-hydroxybutyrate-*co*-4-hydroxybutyrate) [P(3HB-*co*-4HB)] copolymers under specific carbon and fermentation conditions [11, 12]. A study by Thakor et al. [13] showed that P(3HB) homopolymer can be obtained from naphthalene by* Comamonas testosteroni* under specific growth requirement. Since *Comamonas* sp. EB172 is a newly characterized strain, it is necessary to monitor the factors affecting fermentation conditions with respect to their growth, PHA production, P(3HV) incorporation in the copolymer, and the physical properties of the polymer produced, especially molecular weight. 

Previously, it has been reported that the molecular weight of the P(3HB) produced from *Azotobacter chroococcum* 6B was reduced by eightfold by increasing the carbon to the nitrogen ratio [14]. In another report, Quagliano et al. [15] discussed the effect on the chain transfer agent present in the medium on the molecular weight of the P(3HB). Another study reported that the amount of polyhydroxyalkanoate synthase played a key role in controlling the molecular weight and the polydispersity of the polymer [16]. On the other hand, Dennis et al. [17] claimed that, in vivo, the molecular weight would not be a sole function of PHA synthase activity but of the relation between PHA synthase activity and substrate availability. From previous studies, there are several factors affecting the molecular weight and other properties of the PHA; however, reports on PHA properties from *Comamonas* sp. are very limited. Thus, the present study is aimed at determining the effect of carbon to nitrogen ratio and organic nitrogen source on the cell growth and P(3HB-*co*-3HV) production by *Comamonas* sp. EB172 in 2 L fermenter by a pH-stat fed-batch cultivation using technical grade VFAs. The effect of nitrogen source on the molecular weight and mechanical properties of the copolymer produced is also clarified. 

## 2. Materials and Methods

### 2.1. Bacterial Strain, Cultivation, and pH-Stat Fed-Batch Cultivation Processing in 2 L Fermenter

The inoculum preparation, media composition, and cultivation conditions for *Comamonas* sp. EB172 are similar to those reported previously, unless otherwise stated [10]. The pregrown cells from the growth stage (10% v/v) were transferred into 900 mL of mineral salts medium (MSM) containing 3 g/L of technical grade VFA mixtures. The MSM composition is similar to that previously reported unless otherwise stated [9]. The technical grade VFA solution with the concentration of 500 g/L was prepared from mixtures of acetic, propionic, and butyric acids at a ratio of 5 : 3 : 2 (250 g/L : 150 g/L : 100 g/L) for feeding during pH-stat fed-batch fermentation. The composition of the VFA mixtures mimicked the original ratio of acids obtained from anaerobically treated palm oil mill effluent (POME). The technical grade VFA mixtures (with higher concentration) were used instead of real VFAs from POME (normally produced at lower concentration *∼*100 g/L) in order to increase the cell densities in the fermenter. The initial medium pH was set at pH 7.0 with 2 M NaOH. There were two types of nitrogen sources that were used in this study. (NH_4_)_2_SO_4_ was used as inorganic nitrogen source, and the carbon/nitrogen ratios (C/N) (mol/mol) were set at 5, 15, and 25, respectively. Yeast extract (10-11% of nitrogen in YE, Merck, Germany) was used as organic nitrogen source and supplemented with the VFA mixtures at 20 g/L equivalent to C/N 34.4 (mol/mol). The fermentation processes were conducted in batch mode for 10–12 h before the pH-stat fed-batch process was applied. The pH of the culture broth was maintained at pH 7.0 by feeding in the VFA mixture (500 g/L) with C/N ratio mentioned earlier. Dissolved oxygen tension (DOT) in the fermenter (Sartorius, Germany) was maintained at 40% air saturation using cascade mode by gradually increasing the impeller speed. The cultures were incubated at 30°C for 35 h for all experiments.

### 2.2. Analytical Procedures

#### 2.2.1. Microscopy Observation

Both cell cultures from growth and PHA accumulation stage were subjected to microscopic analysis. Negatively stained cells were subjected to physical morphology such as shape, size, and the presence of flagella. Scanning electron microscope (SEM) was used for observation on the cell's morphology changes during growth and accumulation stage. Thin section of the cells containing PHAs was prepared and observed under the transmission electron microscope (TEM). The preparation of the bacterial cell samples was described elsewhere [18]. 

#### 2.2.2. Volatile Fatty Acids Determination

The volatile fatty acids concentration from the broth media was determined by high-performance liquid chromatography (HPLC) (Shimadzu, LC-10 AS) as being described previously [3]. 

#### 2.2.3. Gas Chromatography Analysis

PHA content and composition of the lyophilized cells were determined by using gas chromatography (Agilent, model 7890A) using ID-BP1 capillary column, 30 × 0.32 × 0.25 *μ*m (SGE). A total of 20–25 mg of lyophilized cells were subjected to methanolysis in the presence of methanol and sulfuric acid in the ratio of 85% : 15% (v/v). The resulting hydroxyacyl methyl esters were then analyzed according to the standard method [19]. 

#### 2.2.4. PHA Extraction

PHA in the cells was extracted using solvent extraction method by using chloroform as solvent. The extracted PHA samples (0.5–1.5 g) were soaked in 50–100 mL chloroform for 24–48 h and until completely dissolved. Films were prepared by a solvent-casting technique from chloroform solutions of the polymer using borosilicate glass Petri dishes (Duran, Germany) as casting surfaces. The films were dried until constant weight in a vacuum at room temperature. All samples were stored at −20°C until further analysis. 

#### 2.2.5. Gel Permeation Chromatography Analysis

Number average molecular weight (*M*_*n*_) and weight average molecular weight (*M*_*w*_) were determined by gel permeation chromatography (GPC). The molecular weight of the polyester samples were measured by size exclusion chromatography (SEC) on a TOSOH HLC-8120 GPC system with a refractive index (RI) detector at 40°C using TOSOH TSKgel Super HM-M column and chloroform as eluent at 0.6 mL/min. The sample (12 mg) was dissolved in chloroform (2 mL), and the solution was filtered through a PTFE membrane filter with pore size 0.45 *μ*m (Millipore, USA) and dispensed into collection vials prior to injection by automatic sampler. The samples were run in triplicates, and average values were recorded. 

#### 2.2.6. Differential Scanning Calorimetry Analysis

The thermal properties of the polymer were determined by differential scanning colorimetry (DSC) (TA Instruments). Approximately, 5–7 mg of copolymer samples was weighed and heated from 20 to 200°C at heating rates 10°C/min and held for 1 min. The first scan was conducted to eliminate the history of polymer properties. The samples were then fast cooled from 200°C to −30°C. The second scan was to reheat the samples to 200°C at the same heating rates, and the second scan was used in evaluating the thermal properties of the copolymers [10]. 

#### 2.2.7. Mechanical Properties Analysis

The PHA films with average 0.25–0.35 mm of thickness and 3.0–3.3 mm of width were prepared from 2.0 g of extracted PHA samples. The samples were cut into dumbbell shape using a dumbbell cutter (Die BS 6476). The thickness of the samples was measured using a thickness gauge. The tensile strength and Young's modulus were determined by using Instron Universal Testing Machine (Model 4301) (USA) at 5 mm/min of crosshead speed. The results obtained from the computer system (using Merlin software), such as stress, strain, and elongation at break, were recorded. The results were expressed as a plot of tensile strength (MPa) and tensile modulus (GPa). Mechanical tensile data were calculated from the stress-strain curves on an average of four specimens.

## 3. Results and Discussion

### 3.1. Microscopy Observation of *Comamonas* sp. EB172 during Growth and Production Stage

**Figure 1:**
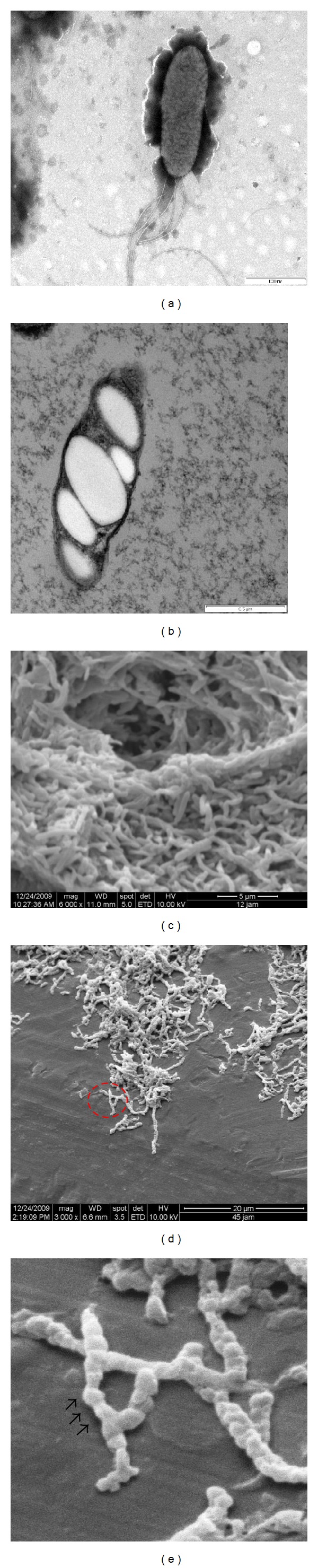
Microscopy observation of *Comamonas* sp. EB172 at various cultivation stages. (a) Negatively stained cells under growth condition, the presence of flagella, and no PHA granules were observed. (b) The presence of PHA granules during PHA accumulation stage viewed under TEM. Scanning electron microscopy of cells (c) during growth stage (magnification 6,000x), (d) during accumulation stage (magnification 3,000x), and (e) zoom out image of cells from red circled in (d); the cells physically changed from smooth rod shape to bulge rod.

The *Comamonas* sp. EB172 is rod in shape, 0.7 to 0.8 by 2.0 to 2.64 *μ*m in size, actively motile by a multipolar flagellum and Gram-negative bacterium (Figure 1(a)). Thin section electron micrograph of the isolates revealed typical PHA granules, which clearly showed the existence of intracellular PHA in the cell during nitrogen depletion condition (Figure 1(b)). It was observed that the morphological shape of the isolates has changed affected by the several packs of granules accumulated in the cell bodies.  Figure 1(c) shows SEM micrographs of the isolate *Comamonas* sp. EB172 under growth stage, and no PHA accumulation was observed. Meanwhile, the shape of the cells obviously changed over 35 h fermentation periods (Figures 1(d)-1(e)) from smooth rod shape to bulge shapes. The accumulation of PHA granules in *Comamonas* sp. EB172 was greatly discussed earlier [18]; however, to our knowledge, the physical changes of the cell observed by the SEM have not been described yet. The size of the cells increased 4-fold in the accumulation stage compared to growth stage. Several *Comamonas* strains have been reported to be capable of accumulating PHA such as *C. testosteroni*, *C. denitrificans*, and *C. composti* [13, 20, 21]. However, none of them showed promising industrial applicability for PHA production. 

### 3.2. Effect of C/N on the Cell Growth and P(3HB-*co*-3HV) Production in the 2 L Fermenter

**Figure 2:**
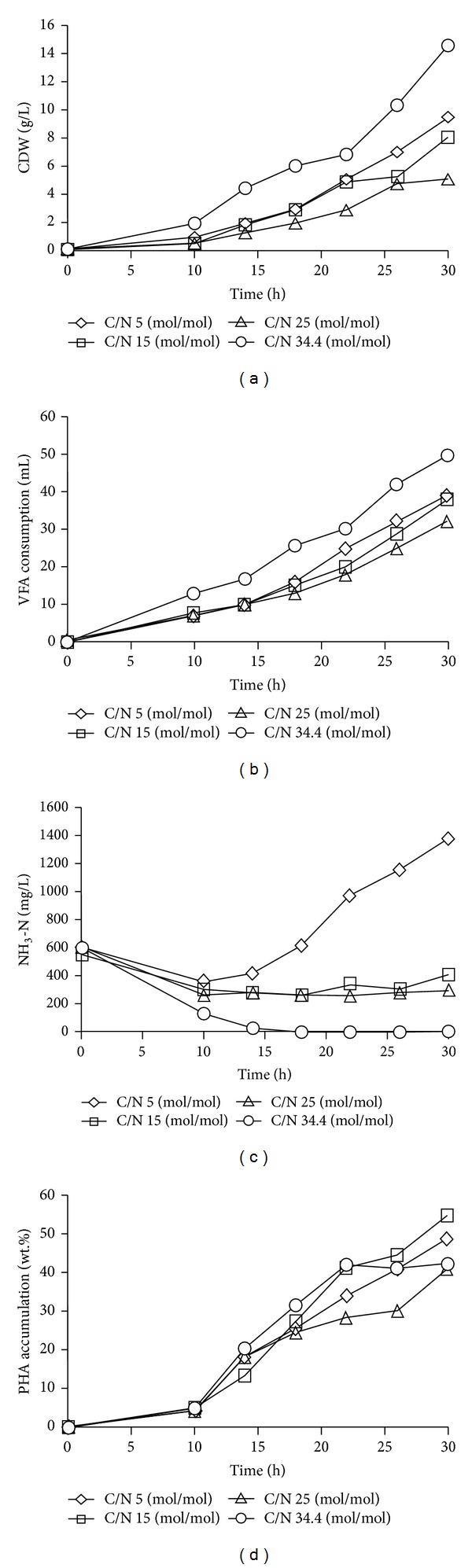
Time course pH-stat fed-batch fermentation of *Comamonas* sp. EB172 in 2 L fermenter (a) CDW (g/L) formation, (b) mixed volatile fatty acids consumed (mL), (c) residual NH_3_–N (mg/L), and (d) PHA accumulation (wt.%) at 30–32 h fermentation period.

Fed-batch cultivation of *Comamonas* sp. EB172 using different C/N ratios was performed, and the results are depicted in Figure 2. Two types of nitrogen sources were used in this study, that is, (NH_4_)_2_SO_4_ and yeast extract. The highest cell dry weight (CDW) formation was recorded at 14.5 g/L when 20 g/L of YE (equivalent to C/N 34.4 (mol/mol)) was supplemented in the feeding solution containing 500 g/L of technical grade VFA mixtures (Figure 2(a)). This was followed by C/N 5  (mol/mol) (9.5 g/L), C/N 15 (mol/mol) (8.1 g/L), and C/N 25 (mol/mol) (5.1 g/L). It was obvious that these results showed that the CDW formation was influenced by the amount of nitrogen (C/N 5, 15, and 25 (mol/mol) from (NH_4_)_2_SO_4_) supplemented into the medium. In contrast, *Comamonas* sp. EB172 showed the highest CDW formation when supplemented with yeast extract even though nitrogen supplementation was limited (C/N 34.4 (mol/mol)). The VFA uptake profiles of all experiments are shown in Figure 2(b). Approximately, 40 mL (*≈*20 g) of VFA was consumed when C/N 34.4 was applied to fermentation process. It was observed that the VFA uptake was correlated to the CDW formation. A different trend was observed on the remaining NH_3_–N (mg/L) concentration in the broth solution after 30 h of the fermentation period (Figure 2(c)). NH_3_–N concentration kept accumulating up to 1300 mg/L when C/N 5 (mol/mol) was supplemented to fermentation medium. However, NH_3_–N concentration from C/N 15 and 25 (mol/mol) was maintained at values 200–400 mg/L. The NH_3_–N concentration was low when YE was used as nitrogen source and totally exhausted towards 17 h of the fermentation period. The residual NH_3_–N content in the fermentation broth is closely related to the CDW (Figure 2(a)) whereby increased cell concentration caused a decrement in NH_3_–N content. This can be explained by the consumption of nitrogen by the cells. The residual NH_3_–N kept accumulating in the medium supplemented with C/N 5 (mol/mol) due to the high nitrogen content in the feed solution and exceeded the optimum requirement from microbial uptake. On the other hand, PHA accumulation was detected as early as 10 h but was considered low (less than 10 wt.%) in the log phase during the growth stage. The highest PHA accumulation was recorded at 53 (wt.%) when C/N 15 (mol/mol) feeding solution was fed into the bioreactor (Figure 2(d)). This was followed by C/N 5 (48 wt.%), YE (43 wt.%), and C/N 25 (40 wt.%). From the results obtained, it is suggested that *Comamonas* sp. EB172 was a growth associate PHA producer as PHA was accumulated during growth stage. This was supported by the high amount of remaining NH_3_–N concentration when C/N 5 feeding solution was used. Other factors such as oxygen limitation will also trigger the accumulation of PHA [13, 22]. Our finding from the shake flask study showed that oxygen limitation has increased the accumulation of PHA by *Comamonas* sp. EB172 (unpublished data). Thus nitrogen and oxygen limitation factors may influence mechanism of polymerization of biopolymers in this microorganism. 

 Optimization of carbon to nitrogen (C/N) is necessary in order to monitor the requirement of growth and PHA accumulation in bacterial cells. Prior to this study, several inorganic and organic nitrogen sources were tested in shake flasks experiment. From the results obtained, (NH_4_)_2_SO_4_ and yeast extract were the best inorganic and organic nitrogen sources, respectively, for PHA accumulation (unpublished data). Furthermore, our previous reports have shown that C/N 30 (mol/mol) was the best for accumulation of PHB but no significant difference on the cells formation (using (NH_4_)_2_SO_4_ as source of nitrogen) [9]. Thus yeast extract was used in order to increase the cells production as well as accumulation of PHA. Several studies have shown that suitable C/N ratio is crucial for obtaining optimal growth and PHA production yield [22–24]. Feeding solution with C/N 10 was fed at the first stage of growth and was followed with C/N 50 to promote accumulation of P(3HB-*co*-3HV) by *Alcaligenes eutrophus* using propionic acid and resulted in 64 g/L of CDW and 58 (wt.%) of accumulation, respectively [23]. *Comamonas* sp. EB172 seems to accumulate PHA during growth stage; thus it may be grouped in the same group as *Azotobacter beijerinckii* and* Alcaligenes latus,* which are known to be the growth associated PHA producers [22]. Even though the accumulation of PHA by *Comamonas* sp. EB172 was not restricted under nitrogen-rich medium, the introduction of nitrogen limitation in the second stage of a fermentation period may enhance the PHA accumulation. Wang and Lee [24] reported that P(3HB) accumulation was enhanced by 27% in *A. latus* when nitrogen limitation was imposed to culture medium. C/N ratio is indeed an important factor in order to obtain higher biomass formation and PHA accumulation as proven by the results from this study. Our current results showed that up to 14.6 g/L of cells could be obtained from 0.4 g/L inocula, which is better than our previous report (9.6 g/L of cells from 1 g/L of inocula) [10]. This finding shows the highest cell dry biomass obtained so far from a genus of *Comamonas* with the ability to accumulate PHA. 

### 3.3. Physical Properties of P(3HB-*co*-3HV) Copolymer Produced

Table 1 shows PHA yield, number average molecular weight (*M*_*n*_), polydispersity index, and mechanical properties of polymers produced using different nitrogen sources. The highest conversion (0.25 g/g) of the volatile fatty acids to PHA was recorded when YE was used as the organic nitrogen source. The results suggested that YE can be a suitable organic nitrogen sources for PHA accumulation in *Comamonas* sp. EB172. The *M*_*n*_ and PDI (*M*_*w*_/*M*_*n*_) recorded for this study ranged from 622 to 838 × 10^3^ Da and 1.8 to 2.1, respectively. From Table 1, it is seen that the *M*_*n*_ of the copolymer varied with the concentration and types of nitrogen source used for the fermentation. The polymer obtained from fermentation with YE as the organic nitrogen source resulted in polymer with the highest molecular weight value. From the results, it can be concluded that the *M*_*n*_ of the copolymer is correlated with the bacterial growth and copolymer yield. This is in agreement with Quagliano and Miyazaki [14] whereby the reduction in the molecular mass of P(3HB) produced was as a result of the low biomass achieved during fermentation. Overall, the use of YE as the nitrogen source stimulated the bacterial growth (Figure 1(a)), PHA yield, and *M*_*n*_ of the copolymer (Table 1). Enhanced bacterial growth is expected when YE is used as the nitrogen source since YE is a complex medium that contains growth precursors. This may increase the cells metabolism and cause the provision of higher substrate levels to the acetoacetyl-CoA reductase, which, resulted in higher production D-3-hydroxyacyl-CoA and hence higher molecular weights of copolymer being produced [17]. This is supported by the yield of PHA (*Y*_*p*/*s*_) obtained during fermentation (Table 1) whereby it is seen clearly that there is positive correlation between *Y*_*p*/*s*_ and the *M*_*n*_ of the copolymer. The high yield of the copolymer produced is a direct consequence of the high production of P(3HB-*co*-3HV) in the cell, which is believed to be contributed by the increased cell metabolism when YE was used as the nitrogen source. We previously reported that the *M*_*n*_ and PDI for P(3HB-*co*-3HV) produced from *Comamonas* sp. EB172 were in the range of 153–412 × 10^3^ Da and 2.2–2.6, respectively [10]. Our present study shows that it is possible to regulate *M*_*n*_ and PDI of the copolymer by using different types of nitrogen sources. The ability to control the molecular weight of the copolymer is an advantage in polymer processing as it offers a wide range of biopolymer applications. 

Meanwhile, the toughness and flexibility of the P(3HB-*co*-3HV) produced by *Comamonas* sp. EB172 were recorded within the range of 13.2–14.4 MPa and 0.26–0.34 GPa, respectively, sharing similar properties to those of polyethylene (Table 1). The flexibility of the copolymers as shown by Young's modulus values seemed to be influenced by the incorporation of the 3HV monomer unit. The P(3HB-*co*-3HV) containing 8 mol% of the 3HV units showed the highest stiffness (0.34 GPa). The study on the mechanical properties of the copolymer produced is important to provide information about the characteristics of the material prior to its use. P(3HB-*co*-3HV) produced from this study exhibited similar properties to those of petroleum-based polyethylene [25], and this is in agreement with other previous reports [2, 11].

## 4. Conclusion

We may conclude that an acid tolerant* Comamonas* sp. EB172 has shown its ability to produce PHA biopolymer using VFAs derived from POME, making this strain a suitable candidate for industrial application. Our investigation on PHA production by this bacterial strain revealed that CDW, PHA yield (g_PHA_/g_substrate_), 3HV (mol%), molecular weight, and mechanical properties of the copolymer can be controlled by varying carbon to nitrogen ratio (mol/mol) and nitrogen source as well. Overall, the natural ability of the bacterium to utilize VFAs for P(3HB-*co*-3HV) production and the opportunity to regulate the molecular weight and mechanical properties of the copolymer produced should render wide-range applications of PHA biopolymers from *Comamonas* sp. EB 172. Continuous production of PHA from VFAs by activated sludge during anaerobic treatment of palm oil mill effluent should be carried out in the future.

## Figures and Tables

**Table 1:** PHA yield and mechanical and thermal properties of P(3HB-*co*-3HV) copolymer produced by *Comamonas* sp. EB172.

Polymer	Sample	3HV (mol%)^1^	PHA yield, *Y*_*p/s*_ (g/g)	*M*_*n*_ (×10^3^), Da^2^	*M*_*w*_/*M*_*n*_^2^	*T*_*m*_	Tensile (MPa)^3^	Young's modulus (GPa)^3^
P(3HB-*co*-3HV)	C/N 15 (mol/mol)	8	0.19	737	1.9	161	13.2	0.34
	C/N 5 (mol/mol)	7	0.11	622	2.1	162	14.4	0.31
	C/N 34.4 (mol/mol)	6	0.25	838	1.8	157	14.1	0.26

Polyethylene, low density (LDPE)^4^		—	—	—	—	—	13.5	0.30

^1^Determined by GC analysis from lyophilized cells.

^2^Determined by gel permeation chromatography.

^3^Determined by Instron Universal Testing Machine.

^4^Adopted from Alexy et al. [25].
